# Platelet Features and Derivatives in Osteoporosis: A Rational and Systematic Review on the Best Evidence

**DOI:** 10.3390/ijms21051762

**Published:** 2020-03-04

**Authors:** Francesca Salamanna, Melania Maglio, Maria Sartori, Matilde Tschon, Milena Fini

**Affiliations:** Laboratory of Preclinical and Surgical Studies, IRCCS Istituto Ortopedico Rizzoli, Via di Barbiano 1/10, 40136 Bologna, Italy; francesca.salamanna@ior.it (F.S.); maria.sartori@ior.it (M.S.); matilde.tschon@ior.it (M.T.); milena.fini@ior.it (M.F.)

**Keywords:** platelet function, platelet derivatives, osteoporosis, bone

## Abstract

*Background*: With the increase in aging population, the rising prevalence of osteoporosis (OP) has become an important medical issue. Accumulating evidence showed a close relationship between OP and hematopoiesis and emerging proofs revealed that platelets (PLTs), unique blood elements, rich in growth factors (GFs), play a critical role in bone remodeling. The aim of this review was to evaluate how PLT features, size, volume, bioactive GFs released, existing GFs in PLTs and PLT derivatives change and behave during OP. *Methods*: A systematic search was carried out in PubMed, Scopus, Web of Science Core Collection and Cochrane Central Register of Controlled Trials databases to identify preclinical and clinical studies in the last 10 years on PLT function/features and growth factor in PLTs and on PLT derivatives during OP. The methodological quality of included studies was assessed by QUIPS tool for assessing risk of bias in the clinical studies and by the SYRCLE tool for assessing risk of bias in animal studies. *Results*: In the initial search, 2761 studies were obtained, only 47 articles were submitted to complete reading, and 23 articles were selected for the analysis, 13 on PLT function/features and growth factor in PLTs and 10 on PLT derivatives. Risk of bias of almost all animal studies was high, while the in the clinical studies risk of bias was prevalently moderate/low for the most of the studies. The majority of the evaluated studies highlighted a positive correlation between PLT size/volume and bone mineralization and an improvement in bone regeneration ability by using PLTs bioactive GFs and PLT derivatives. *Conclusions*: The application of PLT features as OP markers and of PLT-derived compounds as therapeutic approach to promote bone healing during OP need to be further confirmed to provide clear evidence for the real efficacy of these interventions and to contribute to the clinical translation.

## 1. Introduction

**Figure 1:**
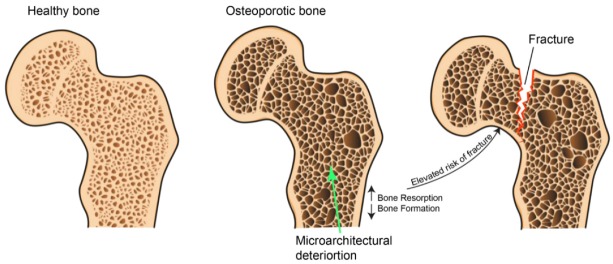
Schematic representation of osteoporosis disease.

Although the bone tissue has the exclusive ability to self-repair and regenerate, in several situations this capability results inadequate or linked to complications. Osteoporosis (OP) is defined by the World Health Organization (WHO) as a “*progressive systemic skeletal disease characterized by low bone mass and microarchitectural deterioration of bone tissue, with a consequent increase in bone fragility and susceptibility to fracture*” [1] (Figure 1).

Affecting about 200 million people in the world, with considerable morbidity and mortality, OP is one of the main epidemics of the 21st century (International Osteoporosis Foundation [2]). Fractures resulting from OP are the main cause of morbidity and mortality, bringing elevated social-economic burden on both families and health care system [3].

Several risk factors, i.e., clinical, medical, behavioral, nutritional and genetic, are related to OP [4]. One of the main causes of OP is the postmenopausal state which involves increased degree of imbalance of bone resorption and formation in favor of bone resorption [5]. Osteoclasts, originating from hematopoietic cells, are mainly responsible for bone resorption. Despite the hematopoietic origin of osteoclasts, the hematological changes occurring during OP are not yet well elucidated. In the last decade, some studies found that platelet (PLT), fragments of cytoplasm derived from the megakaryocytes of the bone marrow, have a critical role in skeletal homeostasis, modulating bone formation and resorption [6,7,8].

**Figure 2:**
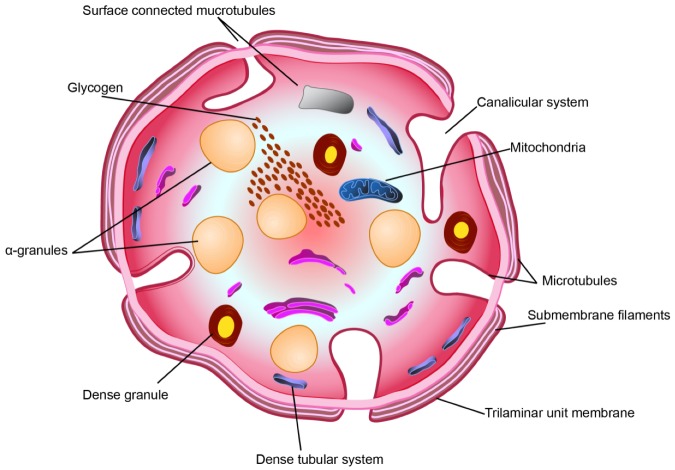
Schematic overview of PLT structure (diagrammatic representation) in the equatorial plane.

PLTs are 2–3 µm in diameter and around their periphery a contractile microtubules ring containing actin and myosin is present. PLTs have several intracellular structures, i.e., lysosomes and two types of granules, dense granule organelles, containing adenosine triphosphate (ATP), adenosine diphosphate (ADP), serotonin, and calcium, and the alpha (α) granules, containing growth factors (GFs), clotting factors, and other proteins (Figure 2) [9]. PLTs also act as a reserve for glycogen [9].

**Figure 3:**
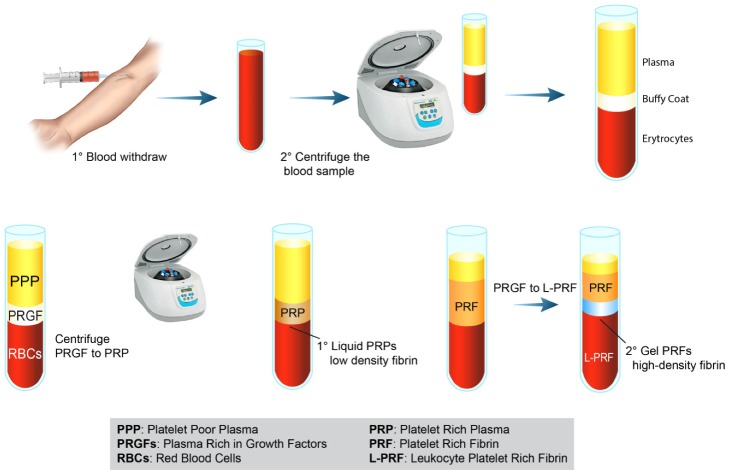
PLT concentrates preparation, types/classes, and illustration/presentation of PLT derivatives. Schematic drawing of the classical preparation protocols of PRP and PRF.

These GFs play a central role in the healing process and tissue regeneration, being used as messengers to regulate various processes [10]. Tissue repair begins with PLT clot formation, activation of the coagulation cascade and PLT degranulation, and release of platelets growth factors (PGFs). These PGFs join to specific target tyrosine growth factor receptors, which then activate intracellular signal transduction pathways [11,12]. Several preclinical and clinical studies highlighted the supportive effect of PLTs on bone formation showing that platelet-derived growth factors (PDGFs) favor bone formation by affecting cell proliferation, chemotaxis differentiation, and extracellular matrix synthesis [13,14]. On the other hand, preclinical in vitro studies showed the role of PLTs in osteoclastogenesis and bone resorption, but the exact mechanism has not been yet proposed [13,14]. These unique biological properties of PLTs emphasize why their derivatives were increasingly used in the clinical scenario to support the healing process in different pathological conditions, including musculoskeletal diseases [15,16,17]. In comparison to the use of a single recombinant GF in high concentrations, the employment of PLT derivatives have the advantage of offering several synergistic GFs able to cooperate in a specific site and for a specific goal. For this reason PLT derivatives (i.e., platelet poor plasma, PPP; platelet-rich plasma, PRP; platelet-rich fibrin, PRF; leucocyte and platelet-rich fibrin, L-PRF) [18] are considered an attractive option for bone tissue regeneration (Figure 3), containing a high concentration of local GFs including PDGF, transforming growth factor (TGF), platelet-derived angiogenesis factor (PDAF), platelet-derived endothelial growth factor (PDEGF), vascular endothelial growth factor (VEGF) and many others able to modulate the regenerative process [19].

It has been observed that PLT derivatives (e.g., PRF, PPP, PRF) improved proliferation and osteogenic activity of bone marrow mesenchymal stem cells (BMSCs) and osteoblasts [20,21,22]. Additionally, in vivo studies have revealed that clots of PLT derivatives, also in combination with different materials/scaffolds, improved bone regeneration by promoting the expression of TGF-β and bone morphogenic protein-2 (BMP-2) [23,24,25,26,27]. Several clinical studies have also applied PLT derivatives, alone and in association with natural and synthetic biomaterials, in patients with different grades of bone defects, reporting improved bone regeneration, early bone formation, bone-depth reduction and more mature bone [28,29,30,31]. However, the exact function of PLTs and its derivatives on bone resorption and bone formation is still complex to understand because of the multifaceted interactions between GFs, inflammatory mediators, and cytokines. Consequently PLTs role and skills during OP and their relationship with bone loss are even more complex to understand and conflicting results have been obtained [32,33,34].

To date, many key questions remain unanswered and controversial, in particular concerning PLT function, size, volume, role of bioactive GFs released and use of PLT derivatives during OP pathogenesis. Thus, we carried out a systematic review in which we wondered: How do PLTs work and what changes occur in their function, features and/or structure (volume, size, number) during OP? How do GFs released by PLTs or GFs existing in PLTs and PLT derivatives “work” during OP? Which are the main derivatives used in OP? and How are they used? In the present systematic review we tried to highlight and answer to these points, attempting to give an up-to-date tool for researchers and clinicians involved in PLT-mediated bone tissue regenerative applications in OP condition.

## 2. Methods

### 2.1. Eligibility Criteria

The PICOS model was used to formulate the questions for this study: (1) studies that considered cells, animals and patients with OP (Population), (2) studies where one of the primary aims were to evaluate PLTs and PLTs derivatives during OP (Interventions), (3) studies that presented a control interventions (Comparisons), (3) studies that reported the effects/functions/roles of PLTs and PLTs derivatives during OP (Outcomes) and (4) preclinical (in vitro and in vivo) and clinical studies (Study design). Studies from 27 July 2009 to 27 July 2019 were included in this review if they met the PICOS criteria.

We excluded studies investigating (1) PLTs functions and/or PLTs derivatives in pathological conditions different from OP, (2) pathological conditions where OP is a bone manifestation of another disease (i.e., diabetes, Gaucher disease, cancer, rheumatic diseases), (3) osteonecrosis of the jaw due to OP therapy, (4) PLT functions and/or PLT derivatives during the administration of drugs active on bone metabolism (e.g., alendronate, zolendronate, denosumab, raloxifene), (5) drug (e.g., glucocorticoid)-induced osteoporosis. Additionally, we excluded case reports, abstracts, editorials, letters, comment to Editor, reviews, meta-analysis, book chapters and articles not written in English.

### 2.2. Information Source and Search Strategies

Our literature review involved a systematic search conducted on 27 July 2019. We performed our review according to the Preferred Reporting Items for Systematic Reviews and Meta-Analyses (PRISMA) statement [35]. The search was carried out on PubMed. MEDLINE, ProQuest, Scopus, Web of Science Core Collection and Cochrane Central Register of Controlled Trials databases to identify preclinical and clinical studies on PLT functions/features and use of PLT derivatives in OP condition. Search was conducted combining the terms “Osteoporosis” AND “Platelets”; for each of these terms, free words and controlled vocabulary specific to each bibliographic database were combined using the operator “OR”. The combination of free-vocabulary and/or MeSH terms for the identification of studies in PubMed/MEDLINE, ProQuest, Scopus, Web of Science Core Collection and Cochrane Central Register of Controlled Trials were reported in Table 1. In addition, reference lists of relevant studies were searched for other potentially appropriate publications.

### 2.3. Study Selection and Data Extraction

Possible relevant articles were screened using title and abstract by one reviewer (FS) and articles that did not meet the inclusion criteria were excluded. After screening the title and abstract, articles were submitted to a public reference manager (Mendeley; “www.mendeley.com”) to eliminate duplicates. Subsequently, the remaining full text articles were retrieved and examined by two reviewers (FS, MM). Any disagreement was resolved through discussion until a consensus was reached, or with the involvement of a third reviewer (MF).

Data from the retrieved studies were tabulated taking into consideration studies that evaluated PLT functions/features and growth factor in PLTs (Table 2) during OP and studies that evaluated PLT derivatives in OP (Table 3). Each table was split-up based on preclinical and clinical studies. We extracted the following data from the articles on PLT functions/features and growth factor in PLTs during OP: Reference, Aim, Study design, Methodology, Platelet function, Link between platelet and OP, Main results (Table 2). The extracted data for the studies on PLT derivatives in OP were: Reference, Aim, Study type, Platelet formulation, Platelets concentration, White blood cells content, Activation method, Platelet application, Experimental design, Main results.

### 2.4. Assessment of Methodological Quality

Two reviewers (FS and MM) independently assessed the methodological quality of selected studies (Table 4 and Table 5). In case of disagreement, they attempted to reach consensus; if this failed, a third reviewer (MF) made the final decision. The methodological quality of the clinical studies was assed using the Quality in Prognosis Studies (QUIPS) tool [36,37]. Studies were assessed on six domains: study participation, study attrition, prognostic factor measurement, outcome measurement, study confounding, and statistical analysis and reporting. Methodological quality appraisal of included in vivo studies was performed according to the Systematic Review Centre for Laboratory Animal Experimentation (SYRCLE) tool [38], which has been specifically designed to assess the risk of bias of animal studies. We have not assessed risk of bias for in vitro studies because, to our knowledge, no standard quality assessment tool exists for the type of in vitro studies included in this review.

## 3. Results

### 3.1. Study Selection and Characteristics

**Figure 4:**
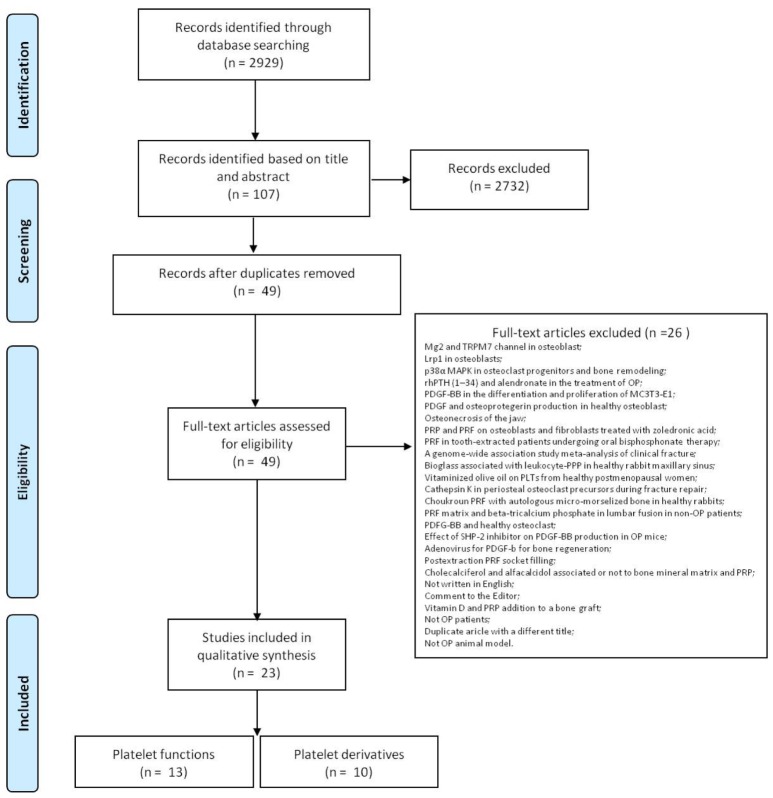
PRISMA flowchart for the selection of studies.

The initial literature search retrieved 2928 studies. Of those, 1169 studies were identified using PubMed/MEDLINE, 167 using ProQuest, 134 using Scopus, 1141 were found in Web of Science Core Collection and 317 using Cochrane Central Register of Controlled Clinical Trials. After screening the title and abstract107 articles were run through Mendeley to eliminate duplicate articles. The resulting 49 complete articles were then reviewed to establish whether the publication met the inclusion criteria and 23 were considered eligible for this review. From the reference lists of the selected articles no additional publications were found. Search strategy and study inclusion and exclusion criteria are detailed in Figure 4.

We divided the extracted data in two tables, taking into consideration studies that evaluated PLT functions/features and growth factor in PLTs during OP (*n* = 13) (Table 2) and studies that used PLT derivatives in OP (*n* = 10) (Table 3). Each table was split-up based on preclinical and clinical studies.

### 3.2. Assessment of Methodological Quality

Risks of bias assessments for each clinical study were summarized in Table 4. The overall risk of bias across studies was low to moderate for the majority of the studies (*n* = 8; 75%), with the exception of two studies that have high risk due the lack of information [33,45], for at least one aspect of study attrition [45], prognostic factor measurement [33] and outcome measurement [45].

Risks of bias assessments for each in vivo study were summarized in Table 5. Risk of bias of animal studies was high for almost all the examined studies. Among the 13 included in vivo studies, three for PLT functions during OP and 10 for PLT derivatives in OP, 10 of them have not declared the method of sequence generation [41,42,49,50,51,52,53,56,57], in one study the method was unclear [55] and in the remaining two studies the method of sequence generation was clearly declared [40,58]. The majority (*n* = 7) of the studies showed that groups were similar concerning baseline characteristics (i.e., age, weight, sex) and two studies showed that allocation was adequately concealed [40,58]. One study reported that animals were housed randomly during the experiment [40] and another reported the blinding of investigators [52]. Only one study reported that the animals were selected at random for outcome assessment [41] and another one reported the blinding of outcome assessors [52]. Almost all the studies included all the animals in the analyses (*n* = 10), reported and detailed the primary outcome (*n* = 12) and were apparently free of other problems that could result in high risk of bias (*n* = 8) (Table 5).

## 4. Study Results

### 4.1. Platelet Functions/Features and Growth Factor in PLTs in Osteoporosis

Articles that evaluated PLT functions/features and growth factor in PLTs during OP were prevalently clinical (*n* = 9), two were in vivo studies, one was both in vivo and clinical and another was exclusively in vitro (Table 2). In this last [39], starting from the evidence of the reduced healing capability of MSCs during OP, a comparison between the effect of BMP-2, BMP-7, parathyroid hormone (PTH) and PDGF on proliferation and osteogenic differentiation of MSCs derived from OP patients was performed. MSCs isolated from trabecular bone showed to be more sensitive to high dose of PDGF-BB, as well of BMP-7 in comparison to the other stimulations, in terms of alkaline phosphatase (ALP) activity and calcium release, in a dose dependent manner. The critical role of the PDGF-BB was also investigated in two in vivo studies [40,41]. Zhang et al. showed that the immobilization of PDGF-BB on titanium nanotube arrays was effective in stimulating osteogenesis both in vitro, on BMSCs isolated from OVX rats, and in an in vivo model of osteointegration in OVX rat femurs [41]. Exploiting the use of a Ctsk^–/–^ mouse model, in which higher levels of PDGF-BB have been found to be secreted by pre-osteoclasts, Xie et al. [40] investigated instead the bone remodeling rate in relationship with angiogenesis stimulation. Results showed that PDGF-BB promotes angiogenesis, recruiting MSCs and endothelial progenitor cells (EPCs) and stimulating CD31hiEmcnhi vessel and bone formation in OVX mice [40]. Additionally, in vitro tube formation assays performed with conditioned medium from pre-osteoclasts and osteoclasts isolated after bone marrow flushing confirmed the results. Always focusing on the PDGF-BB function Tang et al. [42], in an in vivo study on OVX rats and in a prospective clinical study involving young woman, postmenopausal and OP postmenopausal woman, showed that the levels of oestradiol and PDGF-BB correlate with patients age and that the lowest levels are found in the postmenopausal OP cohort.

Several clinical studies evaluated the relationship between PLT and OP status based on Bone Mineral Density (BMD) value considering different cohorts of woman, i.e., healthy, osteopenic and OP [44,46,47]. The investigation on mean PLT volume (MPV) [46,47] and PLT distribution width (PDW) [46] showed that the levels of these markers were reduced in OP patients and that they correlated with BMD T-score. In addition, in a bigger clinical study (175 patients, 72% osteoporotic) it was also seen that MPV inversely correlate with body mass index [46]. On the contrary, performing the same evaluation on the same cohorts of patients, Vural et al. found no differences in MPV and PLT, neither founded any possible relationship between vitamin D levels and MPV [47]. However, D’Amelio et al. [45] evaluating woman with postmenopausal OP and using as control healthy man and woman matched for age and postmenopausal period, showed that PLT vitamin D receptor was less expressed in OP patients. Additionally PLT vitamin D receptor level can be related to the variation of BMD independently form the health status of patients. The lower expression of the receptor also induced a worst response to vitamin D and a consequent increase in PTH levels [45]. In addition, evaluating postmenopausal OP patients, the PLT/lymphocyte ratio was found to correlate with low BMD [33,48], in particular in reference to the femoral and lumbar district [48], which is also related to low vitamin D levels, supporting the hypothesis that inflammation correlates with vitamin D levels [48]. The relationship between peripheral blood cell count and BMD in OP was investigated also by Kim et al. which observed that PLTs count, as well as white and red blood cells counts, correlate with BMD in OP patients [43]. Finally, Kim et al. evaluated the level of plasma PLT activating factor (PAF) in OP woman with radiological evident vertebral fracture. After assessing BMD and serum calcium levels, results showed that PAF levels correlated with the presence of vertebral fracture, as well as with BMD in all sites except for femoral neck, and that PAF levels increase in parallel with ALP levels [34].

### 4.2. Platelet Derivatives in Osteoporosis

All 10 articles on PLT derivatives in OP were about vivo or both in vivo and in vitro studies. With the exception of the study by Rocha et al. [56] that used rabbits submitted to elective ovariosalpingohysterectomy as OP animal model all the other studies employed ovariectomized (OVX) mice and rats to induce OP (Table 3). All studies used PRP as PLT products with the exception of a study which used L-PRF [58]. Six of these studies (60%) also employed a scaffold/biomaterial (calcium phosphate, calcium aluminate/calcium aluminate-melatonin, β-tricalcium phosphate, bovine bone graft, nanoporous TiO_2_, hemostatic sponge) to incorporate the PLTs derivatives and subsequently evaluated bone regeneration in calvaria [51,58], tibia [54,56] and in caudal and lumbar vertebrae [53,57]. Clafshenkel et al. [51] evaluating a calcium aluminate and calcium aluminate-melatonin scaffolds implanted in a critical size calvaria defect of OVX rats, showed that the addition of PRP did not significantly improve degree, intensity and abundance of osteoid tissue mineralization and bone formation in either of the two scaffolds [51]. Differently, Engler-Pinto et al. [58] using the same animal model to evaluate L-PRF alone, blood clot alone, bovine bone graft alone, or a combination of L-PRF with bovine bone graft, showed that the association of L-PRF to bovine bone graft potentiate the bone healing and the production of VEGF, osteocalcin (OCN) and BMP-2/4 [58]. Increased osteogenetic efficiency were also observed evaluating the healing of a long bone defect (tibia) in OVX animals treated with TiO_2_ nanoporous implant associated with PRP [54]. Additionally, it was seen that PRP in association with TiO_2_ not only promoted the osteogenesis but also increased the expression of RUNX2 and COL1 genes and suppressed osteoclastogenesis with increased expression of OPG and decreased levels of RANKL [54]. Despite calvaria defects and long bones defects are the most frequently models used to evaluate bone regeneration and healing during OP, also vertebrae, despite the higher cancellous bone content and the different anatomical and biomechanical properties, were used to evaluate the role of scaffold/biomaterial in association to PRP [53,57]. Two in vivo studies in OVX animals showed that incorporating PRP into calcium phosphate cement [53] and β-TCP sponge [57] accelerated osteoconduction in the caudal [53] and lumbar [57] site, also demonstrating an improvement of the trabecular bone microarchitecture. Additionally, calcium phosphate cements associated to PRP improve the bone mineral density [53] and increase the stiffness of the affected vertebral bodies [57]. Finally, Rocha et al. [56] used a hydrolyzed collagen sponge made from freeze-dried sterile porcine gelatine as carrier for allogenic BMSCs and PRP, alone or in combination, to evaluate the repair of bone failure in tibiae of osteoporotic rabbits secondary to estrogenic deprivation and iatrogenic hypercortisolism [56]. Results suggested that PRP contributed positively to repair of bone failure, but less than the group treated with BMSC and similarly to the association of both [56].

Differently from the above mentioned studies, four studies (40%) used PRP without scaffold/biomaterial to analyze the balance between adipogenesis and osteogenesis in bone regeneration [50], to induce bone regeneration from embryonic fibroblasts [49], to evaluate PRP association to BMSCs [55], to analyze PRP effect in the treatment of OP fractures and to clarify PRP best concentration of use [52]. By using an OVX senescence-accelerated mice (SAMP8) model in which genetically modified NIH3T3 embryonic fibroblasts (pre-differentiated into osteoblast-like cells using PRP) were injected into the bone marrow cavity, an improvement in BMD scores and in the skeletal bone architecture were detected [49]. Using the same animal model it was also seen that PRP alone exerted its action by promoting bone regeneration and suppressing adipogenesis within the marrow [50]. PRP-induced osteogenesis was confirmed by simultaneously up-regulating osteogenesis-promoting genes RUNX2, OPN and OCN and down-regulating adipogenesis regulators such as PPAR-γ2 and leptin in bone marrow cells of PRP treated animals [50]. Allogenic BMSCs in combination with PRP were also used for the treatment of OP bone defects in an OVX rat model showing that bone defects of OVX rats treated with PRP and BMSCs were completely repaired, whereas those treated with PRP or BMSCs alone exhibited slower healing [55]. In addition, higher expression levels of RUNX2, OSX, and OPN were found in rats treated with PRP and BMSCs [55]. Autologous BMSCs cultured with high-, medium-, low-concentration PRP and with PPP from OVX rats were also used to treat fracture healing in an OVX animal model [52]. Results highlighted that the medium-concentration of PRP showed faster healing than the other groups, with a faster bridging of the fracture gaps and higher bridging rate [52].

## 5. Discussion

To date OP management still remains a difficult task for clinicians and based on the progressively increase of aging population the global implications of OP and impaired bone healing are considerable. In the past decade, an increasing number of studies explored the use of new and advanced markers as well as of bioactive factors able to promote bone formation/regeneration during OP [59,60]. Although promising results have been documented both for OP markers and bioactive factors, the available evidence does not yet support their use and further investigation for their clinical use, in particular for PLTs and their derivatives, are mandatory [61]. Thus, the aim of the present systematic review was to evaluate the PLT function, i.e., size, volume, bioactive GFs released, and the usage of their derivatives during OP in order to understand the potential of PLT function as OP markers and the physiopathological mechanisms that underlie the regenerative effects of their derivatives.

In this review, preclinical studies on PLTs function/features and growth factor in PLTs during OP mainly deal with PDGF, a naturally molecule released from the α-granules of PLTs, as part of the clotting process that occurs in response to injury [62]. Homodimer BB constitutes a dimeric glycoprotein of PDGF and is considered the universal PDGF isoform, rendering it the most logical form of the protein to develop as a therapeutic [62]. The ability to simultaneously influence cellular chemotaxis, mitogenesis and angiogenesis gives to PDGF a fundamental role in musculoskeletal repair and regeneration also in OP condition [63]. In this review, PDGF-BB administered in vitro in OP MSCs and in vivo in OVX small animal models respectively stimulated osteogenesis, proliferation and improve angiogenesis and implant osteointegration. In addition, it was found that in OVX animal model, bone marrow levels of PDGF-BB, which was partially produced by preosteoclasts, were drastically decreased. This probably occurred because an increase in mature bone resorption by osteoclasts reduced preosteoclasts and consequently PDGF-BB secretion in OVX animals. Therefore, PDGF-BB is likely mediated by oestrogen in bone metabolism. In fact, it was demonstrated that plasma PDGF-BB levels are maintained by oestrogen in normal young women and play a major role in postmenopausal OP [42]. However, despite numerous studies suggested PDGF-BB as potential therapeutic target during OP, before moving toward the next step, further studies will ascertain the exact mechanisms of PDGF-BB on increasing new bone formation and improving angiogenesis in OP conditions. In addition, clinical studies for dose, delivery site and mode optimization will be mandatory in order to examine the side effects, overall safety and effectiveness of PDGF-BB. In this review the examined clinical studies also found a positive correlation between PLT size, distribution width, volume changes and low BMD due to OP with also a correlation with low levels of PLT vitamin D receptor that underlined a lower ability to respond to vitamin D in OP condition. On the other hand, some other studies demonstrated that PLT size cannot be used as a predictive marker of vitamin D status and BMD during OP. However, as reported by Varol et al. [64] accurate measurements of PLT count and volume are fundamental factors for diagnostic, therapeutic, and research purposes, thus to avoid artefactual results. Unfortunately, not all the studies analyzed detail the procedures used to obtain PLT count and volume and not all used a standardized procedure. An additional mechanism always available with routine blood counts, which explains the relation between PLTs and OP, was found in the correlation between PLT/lymphocyte ratio and OP, since PLT/lymphocyte ratio seems to be a discriminative factor for low BMD. The possibility to exploit data related to PLT size, distribution width, volume changes and PLT/lymphocyte ratio, obtained from a simple and routine investigation, to diagnose and correlate a specific pathological condition is undoubtedly fascinating. However, despite in this review the clinical studies suggested that these parameters may be used as potential OP predictors, a consensus has not been reached and there are still limited results. To date, these aspects strongly restrict the clinical translation and further studies, including larger patient groups, are mandatory and could allow identifying a subset of patients who are at greater risk for developing OP and who may benefit from early screening, intervention, and additional research.

Another key question concerning PLTs regards the role and use of their derivatives during OP. The rationale for PLT derivatives use in bone healing process is due to the abundance and accessibility of key GFs and other signaling molecules in PLTs [65,66,67]. To date, PLT derivatives have been used for the improvement of bone fracture healing, such as common fracture healing, diabetic fracture healing, and nonunion [16,68]. Although not yet definite, it appears that most research supports a positive role for PLT derivatives in bone regeneration. However, the majority of these studies were done in non-OP condition and consequently it is not clear whether the impact of PLT derivatives would be compromised by OP. Thus, in the present review we searched preclinical and clinical studies on PLT derivatives employed in OP condition. However, our search strategy provided only preclinical studies and this is probably due to the fact that the use PLTs derivatives in OP still requires a better understanding of the physiopathological mechanisms that underlie their real regenerative effects. All preclinical studies examined in this review used PRP as PLT products with the exception of one study which used L-PRF and most of them used a scaffold/biomaterial to incorporate the PLTs derivatives. Most of the examined studies demonstrated that PRP improve overall bone quality in OP animal models by promoting osteogenesis while suppressing adipogenesis in bone marrow. Moreover, PRP seems to stimulate the differentiation of embryonic fibroblasts into osteoblast-like cells; the transplantation of these PRP-treated cells also significantly improved bone architecture in OP animal models. It has also been demonstrated that PRP treatment combined with BMSCs may enhance the formation of new bone. However, a minority of studies (2/10) reported that the use of PRP associated to a scaffold and/or to BMSCs did not improve degree, intensity, mineralization and bone formation. Thus, in spite of numerous experimental evidences showed in this review, the use of PLT derivatives during OP is still subject of controversy also considering the high risk of bias of most animal studies. Several explanations for this dispute could be first of all due to the different interval between implantation and investigation as well as to the volume of whole blood and final volume of PLT derivative, final PLT and GFs concentration, methods that PLT derivatives is produced, activator agents, presence or absence of leukocytes and red blood cells, the origin of platetelet derivative used (autologous, allogeneic or xenogeneic). Additionally, specific factors associated to the surgical approach, i.e., size of the bone defect, type and nature of the bone implant, bone graft substitute and bone fixation device could also affect the efficacy. Thus, despite the use of PLT derivatives increased in the past years mainly due to the easy use and biosafety that facilitates the translation in humans, to date further research should be performed to fully reveal the characteristics of the relationship between PLT derivatives and OP. These researches would be of fundamental importance as they would allow a rapid clinical translation of the PLT derivatives in the clinical theatre, leading to an improvement in the patient’s quality of life and a reduction in the ever-increasing financial burden for governments and society due to OP.

## 6. Conclusions

Given the fundamental role of PLT features (size, volume, width distribution, GFs released and growth factor that exists in the PLTs) and PLT derivatives in musculoskeletal repair and regeneration, their future role in OP is expected to expand. Additional researches are under way to further improve our understanding on PLT as markers for OP and on PLT derivatives as therapeutic treatment to enhance bone healing and control inflammation during OP. These future investigations will hopefully continue to shed more light on how PLTs could best used to further improve the outcomes of OP patients in the clinical *scenario*.

## Figures and Tables

**Table 1:** Search terms used in the PubMed, ProQuest, Scopus, Web of Science Core Collection and Cochrane Central Register of Controlled Trials.

Database	Search Items
PubMed	((((((“blood platelets”[MeSH Terms] OR (“blood”[All Fields] AND “platelets”[All Fields]) OR “blood platelets”[All Fields] OR “platelet”[All Fields]) OR (“blood platelets”[MeSH Terms] OR (“blood”[All Fields] AND “platelets”[All Fields]) OR “blood platelets”[All Fields] OR “platelets”[All Fields])) OR ((“blood platelets”[MeSH Terms] OR (“blood”[All Fields] AND “platelets”[All Fields]) OR “blood platelets”[All Fields] OR “platelet”[All Fields]) AND functions[All Fields])) OR ((“blood platelets”[MeSH Terms] OR (“blood”[All Fields] AND “platelets”[All Fields]) OR “blood platelets”[All Fields] OR “platelet”[All Fields]) AND (“Changes”[Journal] OR “changes”[All Fields]))) OR ((“blood platelets”[MeSH Terms] OR (“blood”[All Fields] AND “platelets”[All Fields]) OR “blood platelets”[All Fields] OR “platelet”[All Fields]) AND (“Structure”[Journal] OR “structure”[All Fields]))) OR ((“blood platelets”[MeSH Terms] OR (“blood”[All Fields] AND “platelets”[All Fields]) OR “blood platelets”[All Fields] OR “platelet”[All Fields]) AND size[All Fields])) AND ((((((((“osteoporosis, postmenopausal”[MeSH Terms] OR (“osteoporosis”[All Fields] AND “postmenopausal”[All Fields]) OR “postmenopausal osteoporosis”[All Fields] OR “osteoporosis”[All Fields] OR “osteoporosis”[MeSH Terms]) OR (“bone demineralization, pathologic”[MeSH Terms] OR (“bone”[All Fields] AND “demineralization”[All Fields] AND “pathologic”[All Fields]) OR “pathologic bone demineralization”[All Fields] OR (“bone”[All Fields] AND “demineralization”[All Fields]) OR “bone demineralization”[All Fields])) OR (“bone density”[MeSH Terms] OR (“bone”[All Fields] AND “density”[All Fields]) OR “bone density”[All Fields])) OR (“osteoporotic fractures”[MeSH Terms] OR (“osteoporotic”[All Fields] AND “fractures”[All Fields]) OR “osteoporotic fractures”[All Fields])) OR (“bone diseases, metabolic”[MeSH Terms] OR (“bone”[All Fields] AND “diseases”[All Fields] AND “metabolic”[All Fields]) OR “metabolic bone diseases”[All Fields] OR “osteopenia”[All Fields])) OR (“bone diseases, metabolic”[MeSH Terms] OR (“bone”[All Fields] AND “diseases”[All Fields] AND “metabolic”[All Fields]) OR “metabolic bone diseases”[All Fields] OR (“bone”[All Fields] AND “loss”[All Fields]) OR “bone loss”[All Fields])) OR (“bone density”[MeSH Terms] OR (“bone”[All Fields] AND “density”[All Fields]) OR “bone density”[All Fields])) OR bmd[All Fields]) AND (“2009/07/27”[PDAT]: “2019/07/27”[PDAT])
ProQuest	(platelet AND (bdl(1007527) AND pd(20090727-20190727))) AND (osteoporosis AND (bdl(1007527) AND pd(20090727-20190727))) Applied limits: Database: Biological Science Collection British Nursing Database Health Research Premium Collection *Part of the search defined by the query is performed in these databases*. Restricted based on: Database: Biological Science Collection; Health Research Premium Collection; Biological Science Index; MEDLINE^®^; TOXLINE
Web of Science Core Collection	(TS = platelet OR TS = platelets OR TS = platelet functions OR TS = platelet changes OR TS = platelet structure OR TS = platelet size) AND (TS = osteoporosis OR TS = bone demineralization OR TS = bone density OR TS = Osteoporotic Fractures OR TS = osteopenia OR TS = bone loss OR TS = bone density OR TS = bmd)—with Publication Year from 2009 to 2019
Scopus	(TITLE-ABS-KEY (platelet) OR TITLE-ABS-KEY (platelets) OR TITLE-ABS-KEY (platelet AND functions) OR TITLE-ABS-KEY (platelet AND changes) OR TITLE-ABS-KEY (platelet AND structure) OR TITLE-ABS-KEY (platelet AND size) AND TITLE-ABS-KEY (osteoporosis) OR TITLE-ABS-KEY (bone AND demineralization) AND TITLE-ABS-KEY (bone AND density) OR TITLE-ABS-KEY (osteoporotic AND fractures) OR TITLE-ABS-KEY (osteopenia) OR TITLE-ABS-KEY (bone AND loss) OR TITLE-ABS-KEY (bone AND density) OR TITLE-ABS-KEY (bmd) OR TITLE-ABS-KEY (bone AND mass)) AND DOCTYPE (ar) AND PUBYEAR > 2008
Cochrane Central Register of Controlled Trials	((((((platelet) OR platelets) OR platelet functions) OR platelet changes) OR platelet structure) OR platelet size)) AND (((((((((osteoporosis) OR bone demineralization) OR bone density) OR Osteoporotic Fractures) OR osteopenia) OR bone loss) OR bone density) OR bmd) in All Text—with Publication Year from 2009 to 2019

**Table 2:** Preclinical (in vitro and in vivo) and clinical studies on PLT functions/features and growth factor in PLTs during osteoporosis.

Reference	Aim	Study Design	Methodology	Platelet Function	Link Between Platelet and Osteoporosis	Main Results
*In vitro studies*						
Pountos et al. 2010 [39]	Effect of BMP-2, BMP-7, PTH, and PDGF on proliferation and osteogenic differentiation of OP MSCs	MSCs isolated from trabecular bone of 10 OP patients (4 male and 6 female) treated with a 10^6^ range of concentrations (0.001 to100 ng/mL) of PDGF-BB	Functional assays of proliferation and osteogenic differentiation	PDGF-BB	PDGF-BB have a positive effect on osteogenic differentiation of OP MSCs	MSC proliferation stimulated by BMP-7 and PDGF-BB
*In vivo studies*						
Xie et al. 2014 [40]	Role of PDGF-BB in OVX mice	OVX C57BL/6 female mice injected with 1 μg PDGF-BB into the bone marrow cavity	Micro-CT, immunocytochemistry, immunofluorescence and histomorphometry.	PDGF-BB	Local PDGF-BB administration can temporally increase angiogenesis and spatially promote bone formation to couple angiogenesis with osteogenesis in bone modeling and remodeling	↑PDGF-BB concentrations, VEGF concentrations, vessel volume, CD31hi Emcnhi cells, proliferation of endothelial cells in metaphysis, trabecular bone volume, thickness and number, cortical bone thickness, serum osteocalcin concentration in OVX mice treated with PDGF-BB
Zhang et al. 2014 [41]	Effect on osteointegration of nanotube arrays loaded with rhPDGF-BB	OVX rat femur implantation: - oxalic acid-etched titanium rods - titanium rods modified with TiO_2_ nanotube arrays - PDGF group (titanium rods immersed in 100 μg/mL rhPDGF-BB) - PDGF + Vacuum extraction (vacuum pump -PDGF + Vacuum group-for 10 min)	Static and dynamic histomorphometry and biomechanical test	PDGF-BB	Immobilization of rhPDGF-BB on nanotube arrays as implant surface modification strategy in orthopedic applications in osteoporotic patients	rhPDGF-BB immobilized on the nanotube surface ↑ new bone formation and osseointegration
Tang et al. 2017 [42]	Association between low plasma PDGF-BB levels and oestradiol	Sprague–Dawley rat: -Sham -OVX -OVX+oestradiol (100 mg/kg/d) -OVX+PDGF-BB (1mg/3 d/wk)	Plasma oestradiol and PDGF-BB levels measured using ELISA kits	PDGF-BB	Plasma PDGF-BB levels play a major role in OVX rats	↓PDGF-BB levels in OVX rats than SHAM group. Oestradiol replacement ↑plasma PDGF-BB levels, while PDGF-BB systematic treatment not affect plasma estradiol levels
*Clinical studies*						
Kim et al. 2011 [43]	Association between peripheral blood cell (PLT, WBC, RBC) counts BMD	Case-control study 17 OP patients 167 osteopenic patients 154 control subjects	DXA, biochemical parameters	PLTs count	Positive relationship between blood cell counts and BMD	WBC, RBC and PLT counts significantly associated with BMD
Li et al. 2012 [44]	Relationship between PLT count, MPV, and BMD	Case-control study 111 OP patients 171 osteopenic patients 128 control subjects	DXA, biochemical parameters	PLTs count and MPV	MPV negatively correlated with BMD	Negative correlation between MPV and the lumbar and femoral neck BMD. Univariate and multivariate analysis: MPV significantly associated with lumbar spine L2–L4 BMD and femoral neck BMD
D’Amelio et al. 2012 [45]	Correlation between PLTs vitamin D receptor expression and OP	Case-control study 77 postmenopausal OP patients 33 healthy control of childbearing age 49 healthy control men 11 healthy women matched with patients for age and postmenopausal period	DXA, markers of bone metabolism and vitamin D receptor levels	PLTs vitamin D receptor expression	Reduced level of PLT vitamin D receptor is correlate to OP	↓PLTs vitamin D receptor expression in OP patients respect to healthy postmenopausal controls. PLTs vitamin D receptor not influenced by gender. PLTs vitamin D receptor predict 65% of the BMD variation.
Akbal et al. 2014 [32]	Correlation between BMD and MPV and PDW	Case-control study 30 OP patients 30 osteopenic patients 20 control subjects	DXA, full laboratory test	MPV PDW	Significant role of PDW and MPV in the postmenopausal OP development	↓MPV and PDW in OP than the normal BMD patients. PDW positively correlated with FTT and L1–4T scores. Age and PDW independently related to FTT and LTT scores.
Kim et al. 2015 [34]	Association between plasma PAF, OP vertebral fracture and BMD	Case-control study 73 OP patients with vertebral fracture 73 OP patients without vertebral fracture	Radiography, DXA, biochemical parameters, plasma PAF concentration	PAF	Plasma PAF levels inversely correlated with BMD	34.6% ↑ plasma PAF levels in postmenopausal women with vertebral fracture than subjects without vertebral fracture
Aypak et al. 2016 [46]	Correlation between BMD and MPV	Case-control study 126 OP patients 37 osteopenic patients 12 control subjects	DXA, laboratory tests including complete blood count (CBC), calcium, phosphorus, serum 25 hydroxyvitamin D (25OHD), and intact parathormone (iPTH)	MPV	MPV correlated with BMD in postmenopausal OP women.	MPV significantly associated with BMD in normal weight and overweight-obese OP patients.
Tang et al. 2017 [42]	Association between low plasma PDGF-BB levels and oestradiol in postmenopausal OP	Case-control study 28 postmenopausal OP patients 69 control young woman 24 age-matched women	DXA, plasma oestradiol and PDGF-BB levels	PDGF-BB	Plasma PDGF-BB levels maintained by oestrogen in normal young women and play a major role in postmenopausal OP	↓plasma oestradiol and PDGF-BB levels in postmenopausal women, especially in OP patients. PDGF-BB levels were positively correlated with oestradiol levels and inversely correlated with age
Vural et al. 2017 [47]	Correlation between PLT functions, vitamin D and BMD	Case-control study 124 OP patients 151 osteopenic patients 87 control subjects	DXA, biochemical parameters	MPV	No correlation between MPV and OP. MPV considered a less important indicator in serum 25-hydroxyvitamin D levels and OP	No difference in MPV and PLT counts between groups. No correlation between MPV and serum 25-hydroxyvitamin D levels. Correlation between PLT count and lumbar spine (L1–4) T score
Koseoglu et al. 2017 [48]	Correlation between PLT/lymphocyte *ratio* and low BMD in postmenopausal woman	Case-control study 179 OP and osteopenic patients 32 control subjects	DXA, biochemical parameters	PLT/lymphocyte *ratio*	PLT/lymphocyte *ratio* as new inflammatory marker for bone loss and low BMD	↑PLT/lymphocyte *ratio* in OP and osteopenic patients than in the control subjects. Negative correlation between lumbar and femur neck BMD and PLT/lymphocyte *ratio*
Eroglu et al. 2019 [33]	Correlation between PLT/lymphocyte and BMD	Case-control study 48 OP patients 112 osteopenic patients 92 control subjects	DXA, biochemical parameters	PLT/lymphocyte *ratio*	Negative correlation Between PLT/lymphocyte and BMD	↑PLT/lymphocyte *ratio* in OP and osteopenic patients.

MPV: mean platelet volume; PDW platelet distribution width; FTT: femur total T; L1–4T: lumbar 1–4T; PAF: platelet-activating factor; WBC: peripheral blood white blood cell RBC: red blood cell.

**Table 3:** Preclinical studies on PLT derivatives.

Reference	Aim	Study Type	Platelet Formulation	Platelets Concentration	White Blood Cells Content	Activation Method	Platelets Application	Experimental Design	Main Results
Lo et al. 2009 [49]	Transplantation of PRP/NIH3T3-G cells to induced bone regeneration in OP	in vitro and in vivo	Human PRP	NS	NS	Exogenous-bovine thrombin	NIH3T3-G alone, BMCs alone, and NIH3T3 G/BMC co-culture Bone marrow cavity of the tibia	OVX-SAMP8 mice treated with PRP/NIH3T3-G	PRP/NIH3T3-G treatment prevent OP development
Liu et al. 2011 [50]	Balance between adipogenesis and osteogenesis in bone regeneration by PRP for age-related OP	in vitro and in vivo	Human PRP	NS	NS	Exogenous-bovine thrombin	Mouse pre-adipocytes (3T3-L1) and osteoblast cell line (7F2) co-culture Bone marrow cavity of the hind femur	OVX-SAMP8 mice treated with PRP	PRP treatment exert its action promoting bone regeneration and suppressing adipogenesis within the marrow
Clafshenkel et al. 2012 [51]	Incorporation of melatonin and/or PRP into CA scaffolds to enhance bone regeneration in OP	in vivo	OVX rat PRP	NS	NS	NS	Calvaria critical-sized defect	OVX rats treated with CA scaffold with PRP and melatonin, associated or not	PRP not improves bone formation
Chen et al. 2013 [52]	PRP to promote healing of OP fractures	in vitro and in vivo	OVX rat PRP	High: 8.21 ± 0.4 × 10^9^ Medium:2.65±0.2 × 10^9^ Low: 0.85 ± 0.16x10^9^ PPP:8 ± 0.5x10^6^ (PLTs/mL)	NS	Exogenous-thrombin/CaCl_2_	BMSCs culture Femoral fracture	OVX rats treated with high-, medium- and low-concentration PRP and with PPP	Medium-concentration of PRP is the more suitable in promoting fracture healing
Cho et al. 2014[53]	Incorporation of PRP into CPC to enhance bone regeneration in OP	in vivo	OVX rat PRP	4.12 × 10^9^ (PLTs/mL)	NS	NS	Vertebral body critical-size defects	OVX rats treated with CPC associated to PRP	PRP accelerates osteoconduction and improves trabecular bone microarchitecture and BMD
Jiang et al. 2016 [54]	PRP treatment and TiO_2_ nanoporous modification on the stability of titanium implants in OP	in vivo	Human PRP	2 × 10^9^ (PLTs/mL)	NS	Exogenous-calcium enriched batroxobin	Bone marrow cavity of the hind tibia	OVX rats treated with TiO_2_ associated to PRP	PRP treatment improves implant biomechanical stability
Wei et al. 2016 [55]	PRP in combination with BMSCs for the treatment of OP defect	in vivo	Rats PRP	NS	NS	Exogenous-thrombin	Tibia critical size defects defect	OVX rats treated with allogenic BMSC associated to PRP	PRP combined with BMSCs promotes bone defects healing
Rocha et al. 2017 [56]	PRP and MSCs, associated or not, in the repair of bone failures in secondary OP	in vivo	Equine PRP	200 × 10^3^ (PLTs/µL)	NS	NS	Tibia failures	Rabbits submitted to ovariosalpingohysterectomy and hypercortisolism treated with allogeneic BMSCs and PRP, associated or not	PRP contributes positively to the repair of bone failure, but less than the treatment with MSCs and similarly to the association of both
Sakata et al. 2018 [57]	Bone regeneration of OP defects by PRP and β-TCP	in vivo	Rats PRP	NS	NS	NS	Vertebral body critical-size defects	OVX rats treated with β-TCP associated to PRP	PRP associated to β-TCP sponge facilitates bone regeneration in OVX lumbar vertebral bone defect
Engler-Pinto et al. 2019 [58]	L-PRF associated or not with bovine bone graft on the healing of OP bone defects	in vivo	Rats L-PRF	NS	NS	NS	Calvaria critical size defects	OVX rats treated with bovine bone graft associated to PRP	L-PRF clot improves bone formation but less than the use of L-PRF associated to bovine bone graft

NS: not specified; PRP/NIH3T3-G: NIH3T3-G pre-differentiated into osteoblast-like cells using PRP; OVX-SAMP8: ovariectomized senescence-accelerated mice; CA: calcium aluminate; CPC: calcium phosphate cement; β-TCP: β-tricalciumphosphate.

**Table 4:** QUIPS tool for assessing risk of bias in the clinical studies.

Study	QUIPS					
	Study Participation	Study Attrition	Prognostic Factor Measurement	Outcome Measurement	Confounding Measurement and Account	Analysis
*PLT functions/features and growth factor in PLTs during osteoporosis*						
Kim et al.2011 [43]	Low	Low	Moderate	Moderate	Moderate	Low
Li et al. 2012 [44]	Low	Low	Low	Moderate	Low	Low
D’Amelio et al. 2012 [45]	Moderate	High	Moderate	High	Moderate	Moderate
Akbal et al. 2014 [32]	Low	Low	Low	Moderate	Low	Low
Kim et al. 2015 [34]	Low	Low	Low	Low	Low	Low
Aypak et al. 2016 [46]	Low	Low	Low	Moderate	Moderate	Moderate
Tang et al. 2017 [42]	Low	Moderate	Moderate	Moderate	Moderate	Moderate
Vural et al. 2017 [47]	Low	Low	Low	Moderate	Low	Moderate
Koseoglu et al. 2017 [48]	Low	Low	Low	Moderate	Moderate	Low
Eroglu et al. 2019 [33]	Low	Moderate	High	Moderate	Moderate	Low

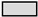
 low (good) indicator, 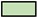 moderate indicator, 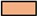 high (bad) indicator.

**Table 5:** SYRCLE’s tool for assessing risk of bias in the in vivo studies.

SYRCLE										
Study	Selection Bias			Performance Bias		Detection Bias		Attrition Bias	Reporting Bias	Other
	Sequence Generation	Baseline Characteristics	Allocation Concealment	Random Housing	Blinding	Random Outcome Assessment	Blinding	Incomplete Outcome Data	Selective Outcome Reporting	Other Sources of Bias
*PLT functions/features and growth factor in PLTs during osteoporosis*										
Xie et al. 2014 [40]	Yes	Yes	Yes	Yes	No	Unclear	No	No	Yes	No
Zhang et al. 2014 [41]	No	Yes	Unclear	Unclear	Unclear	Yes	No	No	Yes	No
Tang et al. 2017 [42]	No	Unclear	Unclear	Unclear	Unclear	Unclear	Unclear	No	Yes	Yes
*PLT derivatives in osteoporosis*										
Lo et al. 2009 [49]	No	Unclear	No	Unclear	Unclear	No	No	No	Yes	No
Liu et al. 2011 [50]	No	No	No	No	No	No	No	No	Unclear	No
Clafshenkel et al. [51]	No	Yes	No	No	No	No	No	Unclear	Yes	Unclear
Chen et al. 2013 [52]	No	Unclear	No	No	Yes	No	Yes	No	Yes	No
Cho et al. 2014 [53]	No	No	No	No	No	No	Unclear	Yes	Yes	Yes
Jiang et al. 2016 [54]	No	Yes	No	No	No	No	No	No	Yes	Unclear
Wei et al. 2016 [55]	Unclear	Yes	Unclear	No	No	No	No	No	Yes	No
Rocha et al. 2017 [56]	No	No	No	No	No	No	No	Unclear	Yes	Yes
Sakata et al. 2018 [57]	No	Yes	Unclear	No	No	No	No	No	Yes	No
Engler-Pinto et al. 2019 [58]	Yes	Yes	Yes	No	No	No	Unclear	No	Yes	No

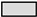
 positive (good) indicator, 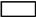 unclear, 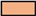 negative (bad) indicator.
